# White Peony (Fermented *Camellia sinensis*) Polyphenols Help Prevent Alcoholic Liver Injury via Antioxidation

**DOI:** 10.3390/antiox8110524

**Published:** 2019-10-31

**Authors:** Yalin Zhou, Fang Tan, Chong Li, Wenfeng Li, Wei Liao, Qin Li, Guohui Qin, Weiwei Liu, Xin Zhao

**Affiliations:** 1 Chongqing Collaborative Innovation Center for Functional Food, Chongqing University of Education, Chongqing 400067, China; zhouyalin@cque.edu.cn (Y.Z.); lichong@cque.edu.cn (C.L.); liaowei@cque.edu.cn (W.L.); qingh@foods.ac.cn (G.Q.); 2 Chongqing Engineering Research Center of Functional Food, Chongqing University of Education, Chongqing 400067, China; 3 Chongqing Engineering Laboratory for Research and Development of Functional Food, Chongqing University of Education, Chongqing 400067, China; 4 College of Biological and Chemical Engineering, Chongqing University of Education, Chongqing 400067, China; 5 Department of Public Health, Our Lady of Fatima University, Valenzuela 838, Philippines; tanfang@foods.ac.cn (F.T.); liqin@foods.ac.cn (Q.L.); 6 School of Life Science and Biotechnology, Yangtze Normal University, Chongqing 408100, China; shanxiliwenfeng@163.com; 7 School of Public Health and Management, Chongqing Medical University, Chongqing 400016, China

**Keywords:** white peony polyphenols, white tea, alcohol, oxidative damage, liver injury

## Abstract

White peony is a type of white tea (*Camellia sinensis*) rich in polyphenols. In this study, polyphenols were extracted from white peony. In vitro experiments showed that white peony polyphenols (WPPs) possess strong free radical scavenging capabilities toward 2,2-Diphenyl-1-picrylhydrazyl (DPPH) and 2,2’-azino-bis(3-ethylbenzothiazoline-6-sulfonic acid) (ABTS). Long-term alcohol gavage was used to induce alcoholic liver injury in mice, and relevant indices of liver injury were examined. WPPs effectively reduced the liver indices of mice with liver injury. The serum levels of aspartate aminotransferase (ATS), alanine aminotransferase (ALT), alkaline phosphatase (ALP), triglycerides (TG), total cholesterol (TC), blood urea nitrogen (BUN), nitric oxide (NO), and malondialdehyde (MDA) were downregulated, while those of albumin (ALB), superoxide dismutase (SOD), catalase (CAT), and glutathione peroxidase (GSH-Px) were upregulated. WPPs also reduced the serum levels of interluekin-6 (IL-6), interluekin-12 (IL-12), tumor necrosis factor-alpha (TNF-α), and interferon-gamma (IFN-γ) in mice with liver injury. Pathology results showed that WPPs reduced alcohol-induced liver cell damage. Quantitative polymerase chain reaction (qPCR) and western blot results revealed that WPPs upregulated the mRNA and protein expressions of neuronal nitric oxide synthase (nNOS), endothelial nitric oxide synthase (eNOS), manganese superoxide dismutase (Mn-SOD), cupro–zinc superoxide dismutase (Cu/Zn-SOD), and CAT and downregulated iNOS expression in the liver of mice with liver injury. WPPs protected against alcoholic liver injury, and this effect was equivalent to that of silymarin. High-performance liquid chromatography revealed that WPPs mainly contained the polyphenols gallic acid, catechinic acid, and hyperoside, which are critical for exerting preventive effects against alcoholic liver injury. Thus, WPPs are high-quality natural products with liver protective effects.

## 1. Introduction

White tea is a slightly fermented tea and is one of the six major teas in China. The raw material of white tea is the bud tip of the tea plant. White tea is light green and covered with frost. The tea soup is also lightly colored; thus, it is called white tea [1]. White peony is mainly produced in regions of Fujian Province, China. Its green leaves contain silver and white hearts in a flower shape. After brewing, the green leaves retain the buds and look like peony blossoms, which suggested the name white peony [2]. White tea possesses bioactivity. It cools the body, moistens the lungs, benefits the blood, has anti-inflammatory and detoxification effects, reduces blood pressure and fat, and decreases tiredness [3,4,5]. The polyphenols in white tea can protect the liver, accelerate the decomposition of the alcohol metabolite acetaldehyde, generate nontoxic substances, and reduce damage to liver cells. White tea can also affect the increased serum lactate dehydrogenase induced by hepatocellular injury, inhibit hepatic M cell collagen fiber formation, help protect the liver, greatly reduce alcohol damage to the liver, and rapidly restore the normal liver function [6].

Alcoholic liver disease is caused by long-term heavy drinking, manifests as fatty liver, and further develops into alcoholic hepatitis, liver fibrosis, and cirrhosis. Its main clinical features include nausea, vomiting, jaundice, hepatomegaly, and tenderness, combined with liver failure and upper gastrointestinal bleeding [7]. Severe drinking may induce extensive hepatocyte necrosis and even liver failure. Alcoholic hepatitis is a common liver disease that severely threatens patients’ health. Alcoholic liver injury results from interactions between inflammatory reactions, oxidative stress, enterogenous endotoxins, and inflammatory mediators that are either directly or indirectly induced by alcohol and its derivatives during metabolic processes [8]. The main metabolic pathway of alcohol is in the liver and requires different subcellular organelles. Alcohol is metabolized by the enzyme alcohol dehydrogenase in the endoplasmic reticulum of the microsomal ethanol oxidation system and by aldehyde oxidase in the mitochondria [9]. The process generates reactive oxygen species (ROS), including superoxide anions, hydroxyl radicals, and hydrogen peroxide. ROS accumulation in the liver leads to functional disorders of the cell membrane, protein and DNA oxidation, and finally hepatocellular injury [10]. Initially, alcohol increases ROS via oxidative stress and induces fat accumulation in the liver. Under the effects of oxidative stress-related lipid peroxidation and inflammatory cytokines, hepatocytes with steatosis undergo a second attack, followed by inflammation, necrosis, and fibrosis [11].

Silymarin is a natural active substance extracted from the dried fruit of the plant silymarin and can protect liver cells from toxic substances, especially alcohol; at the same time, it has a strong antioxidant function, can protect liver cells from free radical damage, and its effect is far better than that of vitamin E; it can also promote the synthesis of proteins, accelerate the production of new liver cells, and repair liver cells [9]. Therefore, silymarin was used as a positive control in this study. White tea possesses antioxidant effects and enhances immunity. In this study, we investigated the effect of white peony (a type of white tea) containing polyphenols on alcoholic liver injury, examining its ability to regulate oxidative stress in mice. The polyphenols present in white peony were also identified to explain the mechanism of action of this tea against alcoholic liver injury.

## 2. Materials and Methods

### 2.1. Extraction of White Tea Polyphenols and Evaluation of the Antioxidant Activity in Vitro

One kilogram of freeze–dried white peony tea (Fuding Jinye Tea Industry Co., Ltd., Fujian, China) was weighed, ground, and divided into 10 samples. An alcohol solution (70% *v/v*) was added to each sample, and extraction was carried out for 4 h in a water bath at 70 °C, followed by filtration. The filtered solution was collected and refiltered through kieselguhr to remove fat-soluble impurities. The filtered solution was collected again and passed through a column of HP20 resin (Cool Chemical Technology Co., Ltd., Beijing, China) to adsorb the polyphenols present in broadleaf holly leaves. Subsequently, a 70% alcohol solution (*v/v*) was used to elute the resin and dissolve the adsorbed polyphenols on the resin. Finally, a white peony polyphenol (WPP) extract was obtained by evaporating the alcohol in a rotary evaporator. The scavenging capability of WPP on 2,2-diphenyl-1-picrylhydrazyl (DPPH) and 2,2’-azino-bis(3-ethylbenzothiazoline-6-sulfonic acid) (ABTS) free radicals was detected in vitro following a previously described method [12].

### 2.2. Animal Experiment

Fifty 6-week-old male specific-pathogen-free Kunming mice (Experimental Animal Center of Chongqing Medical University, Chongqing, China) were acclimated for 1 week and assigned to normal, model, WPP low-concentration (WPPL), WPP high-concentration (WPPH), or silymarin groups (*n* = 10 per group). During the experiment, the mice were allowed to eat and drink freely for 8 weeks. The mice in the WPPL and WPPH groups were fed WPP at 50 mg/kg and 100 mg/kg per day, respectively. Those in the silymarin group were given silymarin by gavage at 100 mg/kg per day. All mice except those in the normal group received 50% alcohol (*v/v*) daily at 0.1 mL/10 g body weight. After 8 weeks, all mice were fasted for 24 h, then sacrificed. Blood was taken from the heart, and the livers were removed for subsequent experiments. The liver tissues were weighed, and the liver index was calculated per a previously established formula (liver index = liver weight/body weight × 100) [13]. The protocol for these experiments was approved by the Ethics Committee of Chongqing Collaborative Innovation Center for Functional Food (201807005B), Chongqing, China.

### 2.3. Determination of Biochemical Indicators in Serum

Blood was centrifuged at 4000 rpm at 4 °C for 10 min (BY-80C centrifugal separator, Beijing Baiyang Medical Instrument Co., Ltd., Beijing, China), then the serum was collected from the supernatant. The serum levels of aspartate aminotransferase (AST), alanine aminotransferase (ALT), alkaline phosphatase (ALP), triglycerides (TG), total cholesterol (TC), blood urea nitrogen (BUN), albumin (ALB), superoxide dismutase (SOD), nitric oxide (NO), catalase (CAT), malondialdehyde (MDA), and glutathione peroxidase (GSH-Px) were measured following the corresponding kits’ instructions (Nanjing Jiancheng Bioengineering Institute, Nanjing, Jiangsu, China). The serum levels of TNF-α, INF-γ, IL-6, and IL-12 were measured via an enzyme-linked immunosorbent assay following the manufacturer’s instructions (Abcam, Cambridge, Massachusetts, USA).

### 2.4. Histological Observations

The liver tissues (0.5 cm^2^) were fixed in 10% formalin for 48 h, dehydrated, cleared in xylene, immersed in wax, embedded, sectioned, and stained with hematoxylin and eosin. The tissue morphology was observed under an optical microscope (BX43 microscope, Olympus, Tokyo, Japan).

### 2.5. RT-qPCR Analysis

The liver tissue was homogenized using a homogenizer, and the total RNA was extracted using TRIzol™ reagent (Thermo Fisher Scientific, Inc., Waltham, MA, USA). The RNA was diluted to 1 μg/μL, then 1 μL of the total RNA solution was reverse-transcribed to obtain a cDNA template following the manufacturer’s instructions (Thermo Fisher Scientific). qPCR was performed using SYBR Green PCR Master Mix (Thermo Fisher Scientific). The upstream and downstream primers used are shown in Table 1 (Thermo Fisher Scientific). qPCR was performed for 40 cycles using the following parameters: 95 °C for 60 s, followed by 95 °C for 15 s, 55 °C for 30 s, and 72 °C for 35 s (SteponePlus, Thermo Fisher Scientific). Glyceraldehyde-3-phosphate dehydrogenase (GAPDH) was used as an internal reference. The 2 ^−ΔΔ^Ct method was used to calculate relative gene expressions [13].

### 2.6. Western Blot

The liver tissue samples (100 mg) were homogenized with 1 mL RIPA (radio immunoprecipitation assay) buffer and 10 mL phenylmethylsulfonyl fluoride (PMSF) (Thermo Fisher Scientific) using a homogenizer at 12,000 r/min at 4 °C for 5 min. The homogenate was centrifuged at 12,000 r/min at 4 °C for 15 min. The middle protein layer was collected, and the proteins were quantitatively measured via the bicinchoninic acid (BCA) method. The samples were diluted at 50 µg/mL, mixed with sample buffer at a ratio of 4:1, heated at 100 °C for 5 min, and loaded into a gel. The separation gel and stacking gel were prepared using acrylamide, stacking buffer, distilled water, 10% ammonium persulfate (APS), and N,N,N’,N’-tetramethylethylenediamine (TEMED) (Thermo Fisher Scientific) at a given ratio. A prestained protein ladder was also loaded into the gel. Vertical gel electrophoresis was performed by SDS-PAGE (sodium dodecyl sulphate polyacrylamide gel electrophoresis) (Thermo Fisher Scientific) for 50 min. A polyvinylidene diflboride (PVDF) membrane was activated by methanol for 1 min, then incubated with a 1×TBST (Tris-Buffered saline Tween-20) solution containing 5% skim milk for 1 h after gel transfer. The PVDF membrane was then washed with 1×TBST and incubated with a primary antibody (Thermo Fisher Scientific) solution at 25 °C for 2 h. The PVDF membrane was further washed with 1×TBST 5 times and incubated with a secondary antibody (Thermo Fisher Scientific) solution at 25 °C for 1 h. Supersignal West Pico PLUS was spread on the PVDF membrane for observation via iBright FL1000 (Thermo Fisher Scientific) [13].

### 2.7. HPLC Analysis

Ten milligrams of the WPP sample was weighed, dissolved in 1 mL dimethyl sulfoxide (DMSO), and heated at 60 °C for 30 min in a water bath. The sample was then removed, and 1.5 mL of pure water and 1.5 mL of ethanol (high-performance-liquid-chromatography [HPLC] grade) were added. The sample was heated in a water bath at 60 °C for 10 min, centrifuged at 2000 rpm for 5 min, and filtered through a 0.22 μm filter membrane. The main components and WPP contents were determined via HPLC (UltiMate3000 HPLC System, Thermo Fisher Scientific). Mobile phase A was water containing 0.5% acetic acid, and mobile phase B was acetonitrile. The flow rate was 0.5 mL/min, with a column temperature of 30 °C, and the detection wavelength was 328 nm. The gradient elution conditions were as follows: equilibrium stage was set for 10 min with 12% B (isocratic), then 0–30 min with 12–45% B (linear gradient), 30–35 min with 45–100% B (linear gradient), and 35–40 min with 100% B (isocratic).

### 2.8. Statistical Analysis

All serum and tissues tests were performed in triplicate. The data were averaged and analyzed using Statistical Package for the Social Sciences (SPSS, Chicago, Illinois, IL, USA) software. One-way analysis of variance was used to analyze the data, and *p* < 0.05 was considered statistically significant.

## 3. Results

### 3.1. Antioxidant Effects of WPPs in Vitro

**Figure 1:**
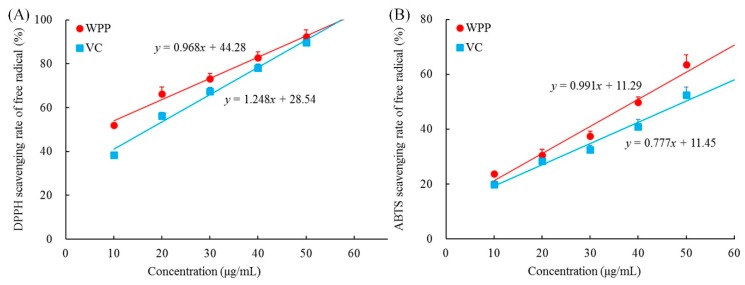
Free radicals scavenging rate of 2,2-Diphenyl-1-picrylhydrazyl (DPPH) (**A**) and 2,2’-azino-bis(3-ethylbenzothiazoline-6-sulfonic acid (ABTS) (**B**). WPP: white peony polyphenols; VC: vitamin C (ascorbic acid).

As shown in Figure 1, the concentration of WPPs ranged from 0 to 50 μg, and the scavenging capacities of WPPs toward DPPH and ABTS increased linearly with their concentrations. With the increase of WPPs concentration, the scavenging effects on DPPH and ABTS radicals were enhanced, and these effects were slightly higher than those of vitamin C (ascorbic acid). It can be seen that WPP could scavenge DPPH and ABTS free radicals in vitro, and the effects were close to those of ascorbic acid, which is known to have good antioxidant properties.

### 3.2. Liver Index

**Figure 2:**
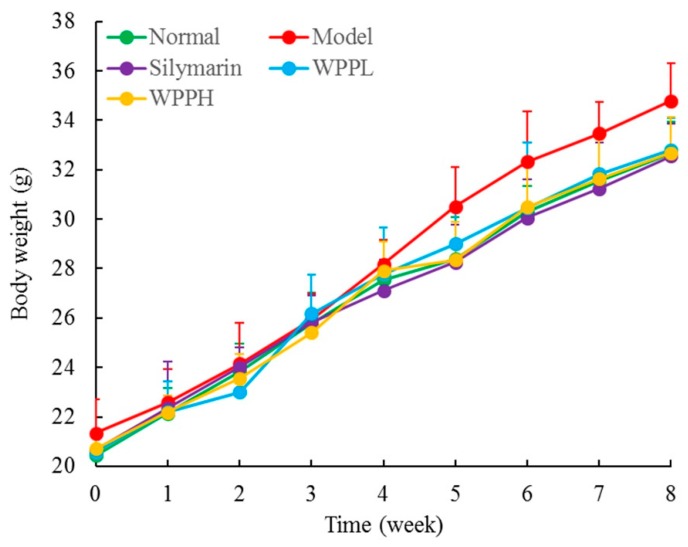
Body weight of mice during the whole experiment. Silymarin: mouse treated with 100 mg/kg silymarin; WPPL: mouse treated with 50 mg/kg white peony polyphenols; WPPH: mouse treated with 100 mg/kg white peony polyphenols.

During the experiment, the weight of the mice in each group increased normally, there was no significant difference (*p* > 0.05) between the weight of the mice in each group, and none of the mice died. (Figure 2). The liver index was the highest in the model group and the lowest in the normal group (Table 2). The liver index of the model group was 2.15 times as high as that of the normal group; after WPP and silymarin treatment, the liver indexes decreased in mice with liver injury, and the effect of a high concentration of WPP (WPPH) was similar to that of silymarin (no significant difference between them, *p* > 0.05); the reduction of the liver index was significantly (*p* < 0.05) greater for WPPH than when administering low-concentration WPP (WPPL).

### 3.3. Liver Function-Related Serum Levels in Mice

The serum ALB levels in the model group were significantly (*p* < 0.05) lower than those in the normal group, but the levels of AST, ALT, ALP, TG, TC, and BUN were all significantly (*p* < 0.05) higher than those in the normal group (Table 3). WPPH reduced the serum levels of AST, ALT, ALP, TG, TC, and BUN and increased the ALB level in mice with liver injury. WPPH was about twice as effective as WPPL, and the effects of WPPH were similar to those of silymarin.

### 3.4. Oxidation-Related Serum Levels in Mice

The SOD, CAT, and GSH-Px enzyme activities in the normal group were significantly higher than those in the other groups *(p <* 0.05), but NO and MDA levels were significantly lower than those in the other groups *(p <* 0.05; Table 4). In the model group, the serum enzymatic activities of SOD, CAT, and GSH-Px were the lowest, while NO and MDA levels were the highest. WPP effectively inhibited the liver injury-induced decreases in SOD, CAT, and GSH-Px activity and increased NO and MDA levels. Moreover, WPPH had a higher effect than WPPL, which was similar to that of silymarin.

### 3.5. Cytokine Serum Levels in Mice

Interluekin-6(IL-6), IL-12, tumor necrosis factor-alpha (TNF-α), and interferon-gamma (IFN-γ) levels in the normal group were significantly lower than those in the other groups *(p <* 0.05), but those in the model group were significantly higher than those of the other groups *(p <* 0.05; Table 5). WPP effectively reduced the serum levels of IL-6, IL-12, TNF-α, and IFN-γ in mice with liver injury, and the effect of WPPH was similar to that of silymarin.

### 3.6. Histological Analyses

**Figure 3:**
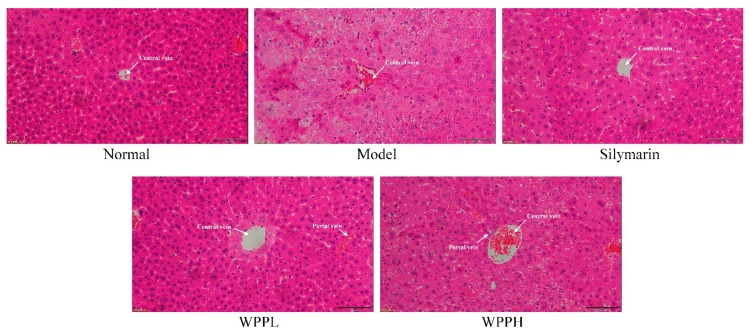
Hematoxylin and eosin staining of hepatic tissues from mice. Magnification 100×. Silymarin: mouse treated with 100 mg/kg silymarin; WPPL: mouse treated with 50 mg/kg white peony polyphenols; WPPH: mouse treated with 100 mg/kg white peony polyphenols.

Liver tissue cells in the normal group were normal in structure, and the hepatocytes were radially distributed around the central vein (Figure 3). In the model group, the cells were nonuniformly arranged, the central veins were irregular, and the cell structure was destroyed, showing much cellular necrosis. Both WPP and silymarin alleviated alcohol-induced hepatocyte necrosis and incomplete morphology in the liver tissues. WPPH had the best effect, which was similar to that of silymarin.

### 3.7. Expression of nNOS, eNOS, and iNOS in the Liver of Mice

**Figure 4:**
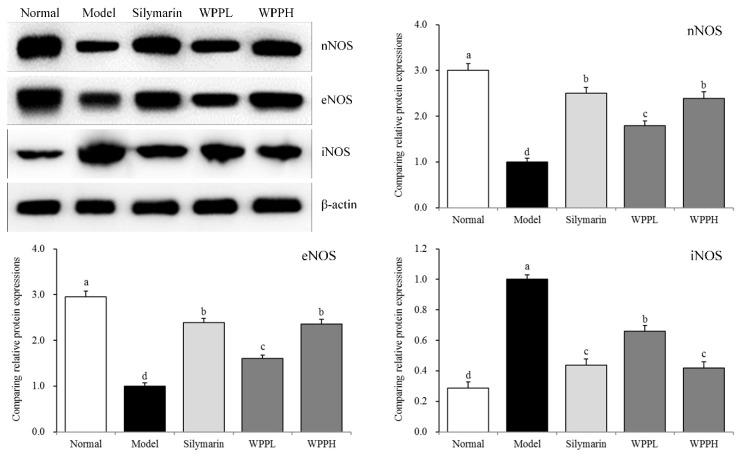
nNOS, eNOS, and iNOS protein expression in the liver tissues of mice. ^a–d^ Using Tukey’s honestly significant different test, there was no significant difference between the two groups with the same superscript (*p* > 0.05), and there was a significant difference between the two groups with different superscript (*p* < 0.05). Silymarin: mouse treated with 100 mg/kg silymarin; WPPL: mouse treated with 50 mg/kg white peony polyphenols; WPPH: mouse treated with 100 mg/kg white peony polyphenols.

nNOS and eNOS mRNA and protein expressions were the highest in liver tissues of the normal group, while iNOS mRNA and protein expressions were the lowest in liver tissues of the normal group (Table 6 and Figure 4). Alcohol decreased the expression of nNOS and eNOS and increasede that of iNOS in the liver. WPP inhibited the enhanced iNOS expression and decreased nNOS and eNOS expressions. The effect of WPPH was similar to that of silymarin.

### 3.8. Expression of Cu-Zn-SOD, Mn-SOD, and CAT in the Liver of Mice

**Figure 5:**
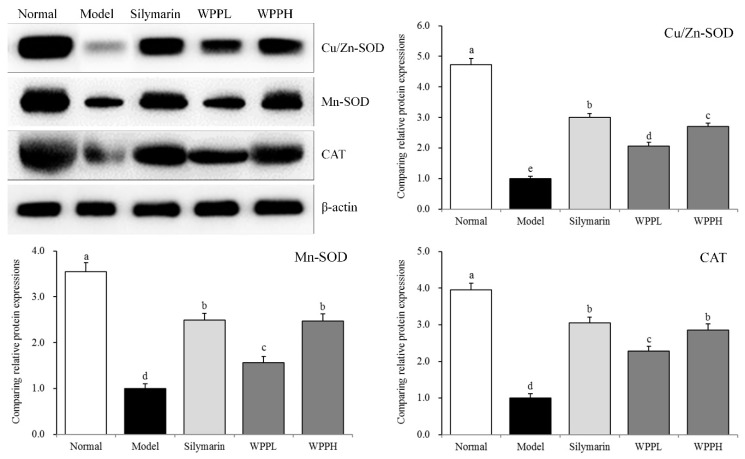
Cu-Zn- SOD, Mn-SOD, and CAT protein expression in the liver tissues of mice. ^a–d^ Using Tukey’s honestly significant different test, there was no significant difference between the two groups with the same superscript (*p* > 0.05), and there was a significant difference between the two groups with different superscript (*p* < 0.05). Silymarin: mouse treated with 100 mg/kg silymarin; WPPL: mouse treated with 50 mg/kg white peony polyphenols; WPPH: mouse treated with 100 mg/kg white peony polyphenols.

Mn-SOD, Cu-Zn-SOD, and CAT mRNA and protein expressions in the liver tissues were the highest in the model group, while iNOS expression was the lowest (Table 7 and Figure 5). Silymarin administered by gavage significantly upregulated Mn-SOD, Cu-Zn-SOD, and CAT expression levels in the livers of mice with liver injury. WPP also upregulated their expression, and the effect of WPPH was greater but did not differ significantly from that of silymarin (*p* > 0.05).

### 3.9. Analysis of the Chemical Composition of WPPs

**Figure 6:**
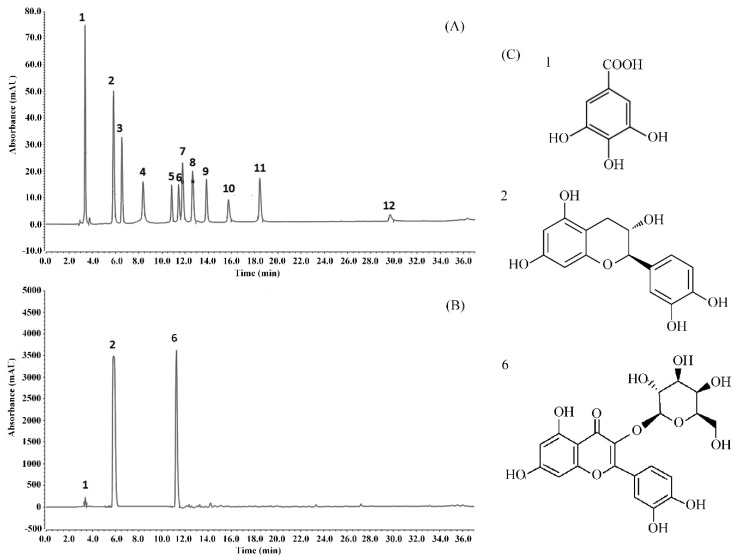
Analysis of the constituents of WPPs determined by HPLC. (**A**) standard chromatogram; (**B**) WPPs chromatograms; (**C**) molecular formulas of the WPPs. 1: gallic acid; 2: catechin; 3: puerarin; 4: gallocatechin gallate; 5: rutin; 6: hyperoside; 7: epicatechin gallate; 8: dihydroquercetin; 9: quercetin; 10: myricetin; 11: thiosulfonate; 12: bata-rhamnetin.

The HPLC results showed that WPPs were rich in gallic acid, catechin, and hypericin with contents of 66.93 mg/g, 427.06 mg/g, and 168.50 mg/g, respectively (Figure 6).

## 4. Discussion

DPPH is a stable radical. ABTS has intermediate stability, and laccase activity can be determined by the rate of laccase oxidation of ABTS [14]. Detecting the scavenging capability of a substance toward DPPH and ABTS can preliminarily determine its antioxidation activity [15]. WPP has good scavenging capability toward DPPH and ABTS, and thus has good antioxidative activity in vitro.

The liver plays an important role in maintaining homeostasis and regulating body functions, including growth and development, disease resistance, and energy supply. With changes in lifestyle and dietary structure as well as increased alcohol production, the proportion of people drinking alcohol is increasing. The incidence of alcohol-related diseases is also increasing yearly. Chronic alcoholism can lead to alcoholic hepatitis, fatty liver, liver cirrhosis, and other alcohol-related diseases [16]. As a classic liver-protecting drug, silymarin has been applied to treat various types of liver injury for a long time. Silymarin has many effects, such as antioxidation, anti-inflammation, immune regulation, and cell regeneration [17]. In this study, it was used as a positive control drug. Liver weight and liver indices were determined to evaluate liver injury induced by carbon tetrachloride. High liver weight and liver indices are manifestations of liver injury [18]. Our study confirmed this and indicated that WPPs downregulated the liver weight and indices in mice with liver injury, and the effects were similar to those of silymarin.

AST is mainly distributed in hepatocytic plasma and mitochondria, and ALT is mainly found in hepatocytic plasma. Hepatocytic necrosis leads to increased ALT and AST levels in the body [19]. Lipid peroxidation in the liver alters hepatocyte membrane permeability, and ALP levels increase sharply [20]. Liver injury may lead to intrahepatic diffusion of fatty acids, resulting in elevated TG content in the liver. TG and TC also reflect lipid peroxidation in the liver, which elevates the TG and TC levels in the body [21]. Decreased liver function can further damage the renal function and increase BUN (a protein metabolite in the blood). Liver injury also affects ALB synthesis, transportation, and release in the body, decreasing ALB levels in the blood [22]. In this study, WPPs also restored the above blood indices to normal values, achieving the same effect as those of silymarin.

Long-term drinking of alcohol leads to oxidation in the body, and the body defends itself against oxidative injury via nonenzymatic and enzymatic antioxidation, for example by regulating and increasing SOD, CAT, and GSH-Px activities [23]. SOD catalyzes the dismutation of superoxide free radicals and scavenges free radicals. CAT and SOD also have synergistic effects which strengthens their effect of scavenging free radicals [24]. CAT is an important antioxidase in the body and can scavenge H_2_O_2_ from the body, thus inhibiting oxidative stress, reducing alcohol-induced oxidation in the body, and inhibiting liver injury [25]. GSH-Px catalyzes hydrogen peroxide decomposition. By catalyzing the reduction of hydrogen peroxide, it can protect cell membranes and avoid cell injury [18]. MDA is a metabolite of lipid peroxidation and is massively accumulated in the body after liver injury [26]. Increases in NO and other oxidative products content can damage phospholipids and proteins on the cell surface and further promote inflammatory exudation and injury [27]. NO reacts with superoxide anion, generating ONOO^-^, aggravates oxidative stress reactions, and leads to cytotoxicity, aggravating liver injury [28]. WPPs upregulated serum SOD, CAT, and GSH-Px levels and downregulated MDA and NO levels, playing a role in counteracting liver injury.

Alcohol can cause oxidation in the body and promote inflammation in the liver, which is reflected by significant increases in the serum levels of IL-6, IL-12, TNF-α, and IFN-γ in mice [29]. IL-6 is secreted by Th2 cells and participates in the humoral immune response. When Th2 levels increase in the body, visceral functions may be damaged [30]. IL-6 can promote differentiation and proliferation of T lymphocytes and antibody generation, alter intracellular G cell activity, upregulate neutrophil function, and enhance inflammatory responses in the body [31]. IL-12 is a strong effective activator of NK cells. Excessive hepatocyte apoptosis and an overly strong immune response aggravates liver injury, partly because of the increased killing function of CD8^+^ T cells by IL-12 [32]. TNF-α binding to the receptor TNF-α R1 on the hepatocyte membrane can lead to the transformation of intracellular double-stranded genomic DNA into oligodeoxynucleotide fragments and promote stem cell apoptosis. TNF-α also aggravates inflammation and liver injury by activating NF-κB [33]. IFN-γ is a proinflammatory cytokine and can enhance the sensitivity of liver cells to TNF-α, rendering liver cells susceptible to damage [34]. Oxidative stress appearing after liver tissue injury can cause an imbalance in inflammatory factors, including IL-6, IL-12, TNF-α, and IFN-γ, in the body, thus increasing their contents in the liver [35]. WPPs can alleviate liver injury by regulating the serum levels of IL-6, IL-12, TNF-α, and IFN-γ in mice with liver injury, and the effect is similar to that of silymarin.

nNOS protects nerve cells and tissues and helps repair damaged tissues [36]. eNOS expression in tissues is relatively stable, and NO generated by eNOS promotes the repair of the liver tissue. eNOS also promotes revascularization further enhancing the repair of damaged liver tissue [37]. After iNOS is activated, the enzyme activity can last a long time, thus releasing a large amount of NO. Low-concentration NO can inhibit gene mutations and enhance the body’s defenses. However, excessive NO stimulates gene mutation and further causes tissue lesions [38]. Oxidative stress leads to aggravated inflammation, resulting in iNOS overexpression, nNOS and eNOS underexpression, and liver injury aggravation [39]. WPPs can upregulate nNOS and eNOS expression, downregulate iNOS expression, and further control the aggravation of inflammation and liver injury caused by oxidative stress, thus exerting a protective effect on the liver.

Mn-SOD and Cu-Zn-SOD are isomers of SOD in the body. Mn-SOD is a SOD free-radical scavenger with Mn^4+^ as its activity center in the mitochondria. Cu–Zn-SOD is another SOD free-radical scavenger that exists in the cytoplasm, having Cu^2+^ and Zn^2+^ as its activity centers [40]. The liver and heart are the organs with the highest number of mitochondria. After alcohol-induced liver injury, Mn-SOD activity significantly declines, which is consistent with the results of our study [34]. Cu-Zn-SOD can eliminate the toxic effect caused by O^2−^•, thus protecting the gastric tissue [41]. Alcohol causes oxidative stress in the body and generates many free radicals. Mn-SOD and Cu–Zn-SOD can inhibit free radicals in the body, helping to prevent liver injury [42]. WPPs can increase the mRNA and protein expressions of Mn-SOD and Cu-Zn-SOD in the liver and further repair liver injury.

As a polyphenol, gallic acid (GA) has strong antioxidation and anti-free radical effects. It can reduce DNA damage in cells caused by oxidation and free radicals and protect tissues [43]. Catechin has a regulatory and antioxidative function in cells, and its antioxidation activity is higher than that of vitamin E, which can efficiently scavenge the free radicals generated in the body and protect cell membranes [44]. Hyperoside is also a strong antioxidant and protects the liver. Its mechanism of action is related to its antioxidative properties, to the normalization of NO levels, and to the improvement of SOD activity [45]. Three substances in WPPs contribute to their protective effects against alcoholic liver injury. Hypericin is uncommon in tea, but present at high levels in white peony, and has biological activity.

## 5. Conclusions

In our study, WPPs reduced the liver indices of mice with liver injury and regulated the related serum levels and the inflammation-related cytokine levels, returning them to normal values. qPCR and western blot further demonstrated that WPPs effectively restored the mRNA and protein expression levels of various liver injury markers in the livers of mice with liver injury. Thus, WPPs have a good attenuating effect on alcoholic liver injury, and their efficacy is similar to that of silymarin. WPPs are a promising natural resource. The possible synergistic effect between the key polyphenols in WPPs should be further investigated.

## Figures and Tables

**Table 1:** Sequences of the primers used for RT-qPCR of the mice liver tissue.

Gene Name	Sequence
nNOS	Forward: 5’-GAATACCAGCCTGATCCATGGAA-3’
	Reverse: 5’-TCCTCCAGGAGGGTGTCCACCGCATG-3’
eNOS	Forward: 5’-TCAGCCATCACAGTGTTCCC-3’
	Reverse: 5’-ATAGCCCGCATAGCGTATCAG-3’
iNOS	Forward: 5’-GTTCTCAGCCCAACAATACAAGA-3’
	Reverse: 5’-GTGGACGGGTCGATGTCAC-3’
Cu–Zn-SOD	Forward: 5’–AACCAGTTGTGTTGTCAGGAC–3’
	Reverse: 5′–CCACCATGTTTCTTAGAGTGAGG–3’
Mn-SOD	Forward: 5’-CAGACCTGCCTTACGACTATGG-3’
	Reverse: 5’-CTCGGTGGCGTTGAGATTGTT-3’
CAT	Forward: 5’-GGAGGCGGGAACCCAATAG-3’
	Reverse: 5’-GTGTGCCATCTCGTCAGTGAA-3’
GAPDH	Forward: 5’-AGGTCGGTGTGAACGGATTTG-3’
	Reverse: 5’-GGGGTCGTTGATGGCAACA-3’

nNOS: neuronal nitric oxide synthase; eNOS: endothelial nitric oxide synthase; iNOS: inducible nitric oxide synthase; Cu–Zn-SOD: cupro–zinc superoxide dismutase; Mn-SOD: manganese superoxide dismutase; CAT: catalase; GAPDH: glyceraldehyde-3-phosphate dehydrogenase.

**Table 2:** Liver index of mice treated with alcohol.

Group	Body Weight	Live Weight	Liver Index
Normal	32.67 ± 1.25 ^a^	1.28 ± 0.11 ^d^	3.92 ± 0.18 ^d^
Model	34.79 ± 1.51 ^a^	2.93 ± 0.48 ^a^	8.42 ± 0.33 ^a^
Silymarin	32.55 ± 1.33 ^a^	1.75 ± 0.27 ^c^	5.38 ± 0.24 ^c^
WPPL	32.81 ± 1.29 ^a^	2.32 ± 0.34 ^b^	7.07 ± 0.29 ^b^
WPPH	32.69 ± 1.44 ^a^	1.88 ± 0.22 ^c^	5.75 ± 0.23 ^c^

“±” for standard deviation (*N* = 10/group). ^a–d^ Using Tukey’s honestly significant different test, there was no significant difference between the two groups with the same superscript (*p* > 0.05), and there was a significant difference between the two groups with different superscripts (*p* < 0.05). Silymarin: mouse treated with 100 mg/kg silymarin; WPPL: mouse treated with 50 mg/kg white peony polyphenols; WPPH: mouse treated with 100 mg/kg white peony polyphenols.

**Table 3:** Levels of AST, ALT, ALP, TG, TC, BUN, and ALB in mice serum (*N* = 10).

Group	ALT (U/L)	AST (U/L)	ALP (K-A)	TG (mmol/L)	TC (mmol/L)	BUN (mg/dL)	ALB (g/dL)
Normal	16.33 ± 1.45 ^d^	10.89 ± 0.38 ^d^	5.80 ± 0.49 ^d^	0.36 ± 0.03 ^e^	1.38 ± 0.15 ^e^	20.18 ± 2.03 ^d^	4.12 ± 0.14 ^a^
Model	74.97 ± 3.62 ^a^	61.28 ± 3.06 ^a^	19.32 ± 1.96 ^a^	2.24 ± 0.26 ^a^	6.37 ± 0.42 ^a^	53.69 ± 3.44 ^a^	1.78 ± 0.10 ^d^
Silymarin	31.25 ± 2.87 ^c^	28.39 ± 2.62 ^c^	10.66 ± 1.21 ^c^	0.92 ± 0.06 ^d^	2.89 ± 0.25 ^d^	32.69 ± 2.71 ^c^	2.86 ± 0.09 ^b^
WPPL	55.69 ± 3.20 ^b^	46.05 ± 2.89 ^b^	15.12 ± 1.02 ^b^	1.79 ± 0.18 ^b^	4.83 ± 0.36 ^b^	41.09 ± 2.42 ^b^	2.39 ± 0.08 ^c^
WPPH	33.17 ± 3.08 ^c^	30.27 ± 2.70 ^c^	11.34 ± 1.19 ^c^	1.20 ± 0.08 ^c^	3.31 ± 0.22 ^c^	33.25 ± 2.16 ^c^	2.77 ± 0.10 ^b^

“±” for standard deviation (*N* = 10/group). ^a–e^ Using Tukey’s honestly significant different test, there was no significant difference between the two groups with the same superscript (*p* > 0.05), and there was a significant difference between the two groups with different superscript (*p* < 0.05). Silymarin: mouse treated with 100 mg/kg silymarin; WPPL: mouse treated with 50 mg/kg white peony polyphenols; WPPH: mouse treated with 100 mg/kg white peony polyphenols. ALT, alanine aminotransferase; AST, aspartate aminotransferase; ALP, alkaline phosphatase; TG, triglycerides; TC, total cholesterol; BUN, blood urea nitrogen; ALB, albumin.

**Table 4:** Levels of SOD, NO, CAT, MDA, and GSH-Px in the serum of mice (*N* = 10).

Group	SOD (U/mL)	NO (µmol/L)	CAT (U/mL)	MDA (µmol/L)	GSH-Px (U/mL)
Normal	148.39 ± 11.35 ^a^	50.33 ± 2.98 ^e^	42.05 ± 2.77 ^a^	5.77 ± 0.52 ^d^	279.52 ± 22.09 ^a^
Model	48.39 ± 4.02 ^d^	169.80 ± 9.23 ^a^	9.83 ± 0.76 ^d^	21.19 ± 1.12 ^a^	93.52 ± 13.71 ^d^
Silymarin	97.25 ± 4.68 ^b^	83.26 ± 4.57 ^d^	28.93 ± 2.10 ^b^	9.89 ± 0.60 ^c^	187.96 ± 18.35 ^b^
WPPL	65.09 ± 4.39 ^c^	133.05 ± 5.09 ^b^	15.32 ± 1.76 ^c^	15.27 ± 0.71 ^b^	127.58 ± 15.50 ^c^
WPPH	95.07 ± 4.26 ^b^	97.37 ± 3.92 ^c^	27.47 ± 1.92 ^b^	10.36 ± 0.48 ^c^	178.07 ± 17.46 ^b^

“±” for standard deviation (*N* = 10/group). ^a–e^ Using Tukey’s honestly significant different test, there was no significant difference between the two groups with the same superscript (*p* > 0.05), and there was a significant difference between the two groups with different superscript (*p* < 0.05). Silymarin: mouse treated with 100 mg/kg silymarin; WPPL: mouse treated with 50 mg/kg white peony polyphenols; WPPH: mouse treated with 100 mg/kg white peony polyphenols. SOD, superoxide dismutase; NO, nitric oxide; CAT, catalase; MDA, malondialdehyde; GSH-Px, glutathione peroxidase.

**Table 5:** Serum levels of IL-6, IL-12, TNF-α, and IFN-γ in mice (*N* = 10).

Group	IL-6 (pg/mL)	IL-12 (pg/mL)	TNF-α (pg/mL)	IFN-γ (pg/mL)
Normal	26.05 ± 2.15 ^e^	188.08 ± 12.59 ^e^	20.85 ± 1.97 ^e^	16.22 ± 1.81 ^e^
Model	238.93 ± 17.73 ^a^	855.67 ± 25.78 ^a^	118.57 ± 7.23 ^a^	89.30 ± 3.92 ^a^
Silymarin	87.36 ± 8.36 ^d^	369.07 ± 18.33 ^d^	51.22 ± 4.36 ^d^	38.77 ± 2.63 ^d^
WPPL	179.20 ± 10.55 ^b^	628.48 ± 20.36 ^b^	88.05 ± 4.93 ^b^	57.08 ± 2.50 ^b^
WPPH	110.25 ± 7.69 ^c^	400.36 ± 19.65 ^c^	67.82 ± 3.06 ^c^	43.17 ± 2.01 ^c^

“±” for standard deviation (*N* = 10/group). ^a–e^ Using Tukey’s honestly significant different test, there was no significant difference between the two groups with the same superscript (*p* > 0.05), and there was a significant difference between the two groups with different superscript (*p* < 0.05). Silymarin: mouse treated with 100 mg/kg silymarin; WPPL: mouse treated with 50 mg/kg white peony polyphenols; WPPH: mouse treated with 100 mg/kg white peony polyphenols. IL-6, interluekin-6; TNF-α, tumor necrosis factor-alpha; IFN-γ; interferon-gamma.

**Table 6:** Neuronal nitric oxide synthase (nNOS), endothelial nitric oxide synthase (eNOS), and inducible nitric oxide synthase (iNOS) mRNA expression in the hepatic tissues of the mice (*N* = 10).

Group	GAPDH	nNOS		eNOS		iNOS	
	Ct value	Ct value	Relative Expression	Ct Value	Relative Expression	Ct Value	Relative Expression
Normal	24.06 ± 0.03 ^a^	24.83 ± 0.08 ^e^	4.20 ± 0.19 ^a^	24.94 ± 0.01 ^d^	3.64 ± 0.06 ^a^	28.86 ± 0.06 ^a^	0.23 ± 0.01 ^e^
Model	24.07 ± 0.01 ^a^	26.90 ± 0.08 ^a^	1.00 ± 0.01 ^d^	26.81 ± 0.09 ^a^	1.00 ± 0.01 ^d^	26.73 ± 0.08 ^e^	1.00 ± 0.01 ^a^
Silymarin	24.03 ± 0.03 ^a^	25.23 ± 0.03 ^d^	3.09 ± 0.14 ^b^	25.47 ± 0.03 ^c^	2.46 ± 0.11 ^b^	27.86 ± 0.04 ^b^	0.45 ± 0.02 ^d^
WPPL	24.02 ± 0.01 ^a^	25.86 ± 0.06 ^b^	1.99 ± 0.10 ^c^	25.94 ± 0.05 ^b^	1.78 ± 0.07 ^c^	27.06 ± 0.05 ^d^	0.78 ± 0.03 ^b^
WPPH	24.04 ± 0.01 ^a^	25.30 ± 0.02 ^c^	2.98 ± 0.09 ^b^	25.51 ± 0.02 ^c^	2.42 ± 0.07 ^b^	27.56 ± 0.05 ^c^	0.55 ± 0.01 ^c^

“±” for standard deviation (*N* = 10/group). ^a–e^ Using Tukey’s honestly significant different test, there was no significant difference between the two groups with the same superscript (*p* > 0.05), and there was a significant difference between the two groups with different superscript (*p* < 0.05). Silymarin: mouse treated with 100 mg/kg silymarin; WPPL: mouse treated with 50 mg/kg white peony polyphenols; WPPH: mouse treated with 100 mg/kg white peony polyphenols.

**Table 7:** Cu–Zn superoxide dismutase (Cu-Zn-SOD), Mn-SOD, and catalase (CAT) mRNA expression in the hepatic tissues of the mice (*N* = 10).

Group	GAPDH	Cu/Zn-SOD		Mn-SOD		CAT	
	Ct Value	Ct Value	Relative Expression	Ct Value	Relative Expression	Ct Value	Relative Expression
Normal	24.06 ± 0.03 ^a^	23.54 ± 0.02 ^e^	6.82 ± 0.22 ^a^	23.91 ± 0.02 ^d^	5.66 ± 0.19 ^a^	24.19 ± 0.05 ^e^	4.66 ± 0.12 ^a^
Model	24.07 ± 0.01 ^a^	26.32 ± 0.07 ^a^	1.00 ± 0.01 ^d^	26.42 ± 0.01 ^a^	1.00 ± 0.01 ^d^	26.42 ± 0.18 ^a^	1.00 ± 0.01 ^d^
Silymarin	24.03 ± 0.03 ^a^	23.78 ± 0.09 ^d^	5.65 ± 0.47 ^b^	24.22 ± 0.03 ^c^	4.45 ± 0.21 ^b^	24.57 ± 0.01 ^d^	3.51 ± 0.07 ^b^
WPPL	24.02 ± 0.01 ^a^	24.02 ± 0.01 ^b^	3.14 ± 0.09 ^c^	24.91 ± 0.04 ^b^	2.77 ± 0.12 ^c^	24.91 ± 0.04 ^b^	2.54 ± 0.15 ^c^
WPPH	24.04 ± 0.01 ^a^	23.95 ± 0.01 ^c^	5.05 ± 0.11 ^b^	24.21 ± 0.01 ^c^	4.52 ± 0.06 ^b^	24.65 ± 0.04 ^c^	3.34 ± 0.13 ^b^

“±” for standard deviation (*N* = 10/group). ^a–e^ Using Tukey’s honestly significant different test, there was no significant difference between the two groups with the same superscript (*p* > 0.05), and there was a significant difference between the two groups with different superscript (*p* < 0.05). Silymarin: mouse treated with 100 mg/kg silymarin; WPPL: mouse treated with 50 mg/kg white peony polyphenols; WPPH: mouse treated with 100 mg/kg white peony polyphenols.

## References

[1] Santana-Rios G., Orner G.A., Amantana A., Provost C., Wu S.Y., Dashwood R.H. (2001). Potent antimutagenic activity of white tea in comparison with green tea in the Salmonella assay. Mutat. Res..

[2] Yang C., Hu Z., Lu M., Li P., Tan J., Chen M., Lv H., Zhu Y., Zhang Y., Guo L. (2018). Application of metabolomics profiling in the analysis of metabolites and taste quality in different subtypes of white tea. Food Res. Int..

[3] Islam M.S. (2011). Effects of the aqueous extract of white tea (Camellia sinensis) in a streptozotocin-induced diabetes model of rats. Phytomedicine.

[4] Almajano M.P., Vila I., Gines S. (2011). Neuroprotective effects of white tea against oxidative stress-induced toxicity in striatal cells. Neurotox. Res..

[5] Espinosa C., López-Jiménez J.A., Pérez-Llamas F., Guardiola F.A., Esteban M.A., Arnao M.B., Zamora S. (2016). Long-term intake of white tea prevents oxidative damage caused by adriamycin in kidney of rats. J. Sci. Food Agric..

[6] Yuan D.S., Li L.M., Yang Z.J., Yue W.J., Zheng J.G. (2009). Effect of liver-protection with different drying temperatures for white tea processing. China Tea.

[7] Teschke R. (2019). Alcoholic liver disease: Current mechanistic aspects with focus on their clinical relevance. Biomedicines.

[8] Wu D., Cederbaum A.I. (2009). Oxidative stress and alcoholic liver disease. Semin. Liver Dis..

[9] Jeong H.M., Kim D.J. (2019). Bone diseases in patients with chronic liver disease. Int. J. Mol. Sci..

[10] Gómez-Bañuelos E., Mukherjee A., Darrah E., Andrade F. (2019). Rheumatoid arthritis-associated mechanisms of *Porphyromonas gingivalis* and *Aggregatibacter actinomycetemcomitans*. J. Clin. Med..

[11] Drescher H.K., Weiskirchen S., Weiskirchen R. (2019). Current status in testing for nonalcoholic fatty liver disease (NAFLD) and nonalcoholic steatohepatitis (NASH). Cells.

[12] Lissi E.A., Modak B., Torres R., Escobar J., Urzua A. (1999). Total antioxidant potential of resinous exudates from Heliotropium species, and a comparison of the ABTS and DPPH methods. Free Radic. Res..

[13] Zhao X., Zhang J., Yi S., Li X., Guo Z., Zhou X., Mu J., Yi R. (2019). *Lactobacillus plantarum* CQPC02 prevents obesity in mice through the PPAR-α signaling pathway. Biomolecules.

[14] Soares D.G., Andreazza A.C., Salvador M. (2003). Sequestering ability of butylated hydroxytoluene, propyl gallate, resveratrol, and vitamins C and E against ABTS, DPPH, and hydroxyl free radicals in chemical and biological systems. J. Agric. Food Chem..

[15] Martysiak-Żurowska D., Wenta W. (2012). A comparison of ABTS and DPPH methods for assessing the total antioxidant capacity of human milk. Acta Sci. Pol. Technol. Aliment..

[16] Gao B., Bataller R. (2011). Alcoholic liver disease: Pathogenesis and new therapeutic targets. Gastroenterology.

[17] Colica C., Boccuto L., Abenavoli L. (2017). Silymarin: An option to treat non-alcoholic fatty liver disease. World J. Gastroenterol..

[18] Wang M., Niu J., Ou L., Deng B., Wang Y., Li S. (2019). Zerumbone protects against carbon tetrachloride (CCl_4_)-induced acute liver injury in mice via inhibiting oxidative stress and the inflammatory response: Involving the TLR4/NF-κB/COX-2 pathway. Molecules.

[19] Iweala E.E.J., Evbakhavbokun W.O., Maduagwu E.N. (2019). Antioxidant and hepatoprotective effect of *Cajanus cajan* in N-nitrosodiethylamine-induced liver damage. Sci. Pharm..

[20] Albasher G., Almeer R., Al-Otibi F.O., Al-Kubaisi N., Mahmoud A.M. (2019). Ameliorative effect of *Beta vulgaris* root extract on chlorpyrifos-induced oxidative stress, inflammation and liver injury in rats. Biomolecules.

[21] Wang R., Yang Z., Zhang J., Mu J., Zhou X., Zhao X. (2019). Liver injury induced by carbon tetrachloride in mice is prevented by the antioxidant capacity of Anji White tea polyphenols. Antioxidants.

[22] Tang B.B., Hu D.H. (2016). Effect of early bedside hemofiltration on systemic inflammatory state as well as liver and kidney function in patients with severe acute pancreatitis. J. Hainan Med. Univ..

[23] Farbiszewski R., Radecka A., Chwiecko M., Holownia A. (1992). The effect of heparegen on antioxidant enzyme activities in ethanol-induced liver injury in rats. Alcohol.

[24] Huang Q.H., Xu L.Q., Liu Y.H., Wu J.Z., Wu X., Lai X.P., Li Y.C., Su Z.R., Chen J.N., Xie Y.L. (2017). Polydatin protects rat liver against ethanol-induced injury: Involvement of CYP2E1/ROS/Nrf2 and TLR4/NF-κB p65 pathway. Evid. Based Complement. Altern. Med..

[25] Li Y.G., Ji D.F., Chen S., Hu G.Y. (2008). Protective effects of sericin protein on alcohol-mediated liver damage in mice. Alcohol Alcohol..

[26] Wang X., Liu M., Zhang C., Li S., Yang Q., Zhang J., Gong Z., Han J., Jia L. (2018). Antioxidant activity and protective effects of enzyme-extracted *Oudemansiella radiata* polysaccharides on alcohol-induced liver injury. Molecules.

[27] Sun X., Wang P., Yao L.P., Wang W., Gao Y.M., Zhang J., Fu Y.J. (2018). Paeonol alleviated acute alcohol-induced liver injury via SIRT1/Nrf2/NF-κB signaling pathway. Environ. Toxicol. Pharmacol..

[28] Clemens M.G. (1999). Nitric oxide in liver injury. Hepatology.

[29] An L., Wang X., Cederbaum A.I. (2012). Cytokines in alcoholic liver disease. Arch. Toxicol..

[30] Eipel C., Hardenberg J., Negendank S., Abshagen K., Vollmar B. (2009). Thrombopoietin limits IL-6 release but fails to attenuate liver injury in two hepatic stress models. Eur. J. Gastroenterol. Hepatol..

[31] Cheng L., Wang J., Li X., Xing Q., Du P., Su L., Wang S. (2011). Interleukin-6 induces Gr-1+CD11b+ myeloid cells to suppress CD8+ T cell-mediated liver injury in mice. PLoS ONE.

[32] Cheng L., Du X., Wang Z., Ju J., Jia M., Huang Q., Xing Q., Xu M., Tan Y., Liu M. (2014). Hyper-IL-15 suppresses metastatic and autochthonous liver cancer by promoting tumour-specific CD8+ T cell responses. J. Hepatol..

[33] Zhou H.C., Wang H., Shi K., Li J.M., Zong Y., Du R. (2019). Hepatoprotective effect of baicalein against acetaminophen-induced acute liver injury in mice. Molecules.

[34] Liu B., Fang Y., Yi R., Zhao X. (2019). Preventive effect of blueberry extract on liver injury induced by carbon tetrachloride in mice. Foods.

[35] Qiao J.Y., Li H.W., Liu F.G., Li Y.C., Tian S., Cao L.H., Hu K., Wu X.X., Miao M.S. (2019). Effects of *Portulaca Oleracea* Extract on Acute Alcoholic Liver Injury of Rats. Molecules.

[36] Alexaki V.I., Charalampopoulos I., Kampa M., Vassalou H., Theodoropoulos P., Stathopoulos E.N., Hatzoglou A., Gravanis A., Castanas E. (2004). Estrogen exerts neuroprotective effects via membrane estrogen receptors and rapid Akt/NOS activation. FASEB J..

[37] Wickman A., Jonsdottir I.H., Bergstrom G., Hedin L. (2002). GH and IGF-I regulate the expression of endothelial nitric oxide synthase (eNOS) in cardiovascular tissues of hypophysectomized female rats. Eur. J. Endocrinol..

[38] Hayashi Y., Abe M., Murai A., Shimizu N., Okamoto I., Katsuragi T., Tanaka K. (2005). Comparison of effects of nitric oxide synthase (NOS) inhibitors on plasma nitrite/nitrate levels and tissue NOS activity in septic organs. Microbiol. Immunol..

[39] Lin H.I., Wang D., Leu F.J., Chen C.F., Chen H.I. (2004). Ischemia and reperfusion of liver induces eNOS and iNOS expression: Effects of a NO donor and NOS inhibitor. Chin. J. Physiol..

[40] Liu B., Li J., Yi R., Mu J., Zhou X., Zhao X. (2019). Preventive effect of alkaloids from *Lotus plumule* on acute liver injury in mice. Foods.

[41] Kanai S., Okano H. (1998). Mechanism of the protective effects of sumac gall extract and gallic acid on the progression of CCl_4_-induced acute liver injury in rats. Am. J. Chin. Med..

[42] Wheeler M.D., Nakagami M., Bradford B.U., Uesugi T., Mason R.P., Connor H.D., Dikalova A., Kadiiska M., Thurman R.G. (2001). Overexpression of manganese superoxide dismutase prevents alcohol-induced liver injury in the rat. J. Biol. Chem..

[43] Sen S., Asokkumar K., Umamaheswari M., Sivashanmugam A.T., Subhadradevi V. (2013). Antiulcerogenic effect of gallic acid in rats and its effect on oxidant and antioxidant parameters in stomach tissue. Indian J. Pharm. Sci..

[44] Rossetto M., Vanzani P., Mattivi F., Lunelli M., Scarpa M., Rigo A. (2002). Synergistic antioxidant effect of catechin and malvidin 3-glucoside on free radical-initiated peroxidation of linoleic acid in micelles. Arch. Biochem. Biophys..

[45] Kim S.J., Um J.Y., Lee J.Y. (2011). Anti-inflammatory activity of hyperoside through the suppression of nuclear factor-κB activation in mouse peritoneal macrophages. Am. J. Chin. Med..

